# Engineering of ‘Purple Embryo Maize’ with a multigene expression system derived from a bidirectional promoter and self‐cleaving 2A peptides

**DOI:** 10.1111/pbi.12883

**Published:** 2018-03-12

**Authors:** Xiaoqing Liu, Wenzhu Yang, Bona Mu, Suzhen Li, Ye Li, Xiaojin Zhou, Chunyi Zhang, Yunliu Fan, Rumei Chen

**Affiliations:** ^1^ Department of Crop Genomics & Genetic Improvement Biotechnology Research Institute Chinese Academy of Agricultural Sciences Beijing China

**Keywords:** anthocyanin biosynthesis, bidirectional promoter, 2A linker peptide, multigene expression, metabolic engineering, transgenic maize

Anthocyanins are polyhydroxy and polymethoxy 2‐phenylbenzopyrylium glucosides that belong to a large group of plant secondary metabolites termed flavonoids. The most common anthocyanidins (anthocyanin aglycones) in higher plants are cyanidin, pelargonidin, peonidin, delphinidin, petunidin and malvidin. Flavonoid biosynthesis has been suggested to occur on the cytoplasmic face of the endoplasmic reticulum (ER), where sequential enzymes of the pathway loosely form a metabolon (Winkel‐Shirley, 1999). Known structural genes along the anthocyanin biosynthesis pathway in maize include chalcone synthase (CHS), chalcone isomerase (CHI), flavanone 3‐hydroxylase (F3H), flavonoid 3′‐hydroxylase (F3′H), dihydroflavonol 4‐reductase (DFR), anthocyanidin synthase (ANS), flavonoid 3‐O‐glucosyltransferase (3UFGT) and glutathione S‐transferase (GST). Known regulators of anthocyanin biosynthesis contain MYC‐like bHLH (basic helix‐loop‐helix) proteins and R2R3‐MYB proteins. MYC‐like bHLH proteins are encoded by the *R* locus (*S*,* Lc* and *Sn*) and *B* locus (*b1*, booster1), whereas MYB regulators are encoded by *c1* (*colorless1*), *pl1* (*purple plant1*) and *p1* (pericarp color1). C1 or PL1 along with R1 or B1 and WD40 Pale Aleurone Color1 (PAC1) form a ternary MYB‐bHLH‐WD40 (MBW) transcription factor complex to regulate the synthesis of anthocyanins at the transcriptional level in maize kernels or vegetative plant parts (Sharma *et al*., 2011). In addition, other regulatory genes include *viviparous1* (*vp1*), *anthocyaninless lethal1* (*anl1*) and *intensifier1* (*in1*). VP1 controls the anthocyanin pathway in maize seed development primarily through activation of the *C1* gene in the aleurone layer (McCarty *et al*., 1989). The IN1 gene product is a repressor that shares homology with R1/B1 and functions as a competitive inhibitor of R1 by binding to C1 (Burr *et al*., 1996).

Crop performance depends on multiple traits and metabolic pathways, and multiple traits must simultaneously be addressed during crop improvement. To meet these broad demands, diverse multigene stacking strategies have been developed to introduce multiple genes or complex metabolic pathways into plants. Sexual crossing, a simple but time‐consuming and labour‐intensive strategy, has been successfully applied in traditional cross‐breeding (hybrid rice) but is powerless in introducing genes from species that are not sexually compatible with the crop of interest. Cotransformation mediated by biolistic gene transfer can simultaneously deliver multiple transgenes but is coupled with the drawbacks of complex genome integration of transgenes and unstable linkage inheritance between generations (Chen *et al*., 1998). Serial retransformation is another approach for stacking multiple transgenes but requires different selection marker genes for each transformation (Qi *et al*., 2004). Assembly of multiple individual cassettes into a T‐DNA vector is a common multigene stacking strategy when using Agrobacterium‐mediated plant genetic transformation (Zhu *et al*., 2017), but the challenges of potential gene silencing caused by repetitious use of the same promoter, large size of the T‐DNA construct and complex recombination steps of multiple genes stacking in a vector may limit its application. 2A peptides found in various viruses and *Impatiens balsamina* are self‐cleaving linker peptides varying from 16 to 20 amino acids in length (Szymczak *et al*., 2004). No matter in animal or plant cells, individual proteins can be released from translated, 2A‐linked, multicistrons by self‐cleaving (Ha *et al*., 2010; Szymczak *et al*., 2004). In this study, we combine the advantages of a bidirectional promoter and self‐cleaving 2A peptides to produce a simple and efficient multigene expression system (MES) that can be used for polyprotein expression and engineering complex metabolic pathways, as we demonstrate by rebuilding the anthocyanin biosynthesis pathway in maize embryos.

Four synthesized basic multicistrons were linked by 2A peptides from different origins: (i) contained four fluorescent protein genes (*GFP*,* YFP*,* CFP* and *mCherry*), (ii) β‐glucuronidase gene (*GUS*), (iii) two phytase genes (*AO* and *CP53*) and (iv) four anthocyanin biosynthesis‐related genes (*ZmBz1*,* ZmBz2*,* ZmC1* and *ZmR2*). Each was then fused to both ends of P_ZmBD1_, which obtained from maize inbred line B73 (Liu *et al*., 2016) to generate multigene expression constructs. Before stable transformation, these constructs were tested by bombardment of HiII maize immature embryos (20 days after pollination). The signal of GFP and GUS located in the 3′ end of each multicistron could be detected, and anthocyanin could be synthesized in the bombarded immature embryos resulting from the expression of *ZmBz1*,* ZmBz2*,* ZmC1* and *ZmR2* located in the 5′ end of each multicistron. These results indicated that these linked genes in long multicistrons could be transiently transcribed, translated and processed into functional products and, furthermore, illustrated that the MES based on an embryo‐specific bidirectional promoter and 2A peptides is feasible.

For further verification, the constructs were introduced into HiII maize via *Agrobacterium*‐mediated transformation to generate stable transgenic maize plants. PCR analysis was carried out with T4 generation to confirm the integration of 11 transgenes and the *bar* selectable marker gene; results suggested that all 12 transgenes are stably integrated in the genome. We also verified the products of transgenes by Basta spraying for *bar*, X‐Gluc staining for *GUS*, fluorescence visualization for *GFP* and anthocyanin biosynthesis for *ZmBz1*,* ZmBz2*,* ZmC1* and *ZmR2* in developing kernels. GUS and GFP signals and anthocyanin biosynthesis were detected in the same regions of embryos and aleurone. These results confirmed that the MES provides a simple and efficient means of delivering multiple genes, which could be utilized in complex metabolic engineering.

In traditional edible grains, anthocyanins accumulate only in two parts with limited biomass, the pericarp and aleurone layer, while the endosperm and embryo lack anthocyanins. Recently, the first anthocyanin‐biofortified rice ‘zijingmi’ was engineered by introducing eight foreign genes involved in anthocyanin biosynthesis into the genome of ZH11 (Zhu *et al*., 2017). Here, for the first time, we engineered ‘Purple embryo maize’ (Figure 1) by changing the spatiotemporal expression characteristics of two or four anthocyanin biosynthesis‐related maize genes using the MES depicted above. Analysis of anthocyanin composition with high‐performance liquid chromatography (HPLC) indicated that cyanidin is the major constituent in transgenic maize kernels, as in PMZ1 (a purple reference maize line), while HiII kernels contain no anthocyanins (Figure 1b/c). Total anthocyanin content of kernels derived from most transgenic events contained more than 200 mg/kg dry weight, with the highest content from three different constructs, pCL1, pCL3 and pCL4, reaching 1035, 847 and 897 mg/kg dry weight, respectively (Figure 1c). To investigate why anthocyanin is not synthesized and accumulated in embryos of HiII, we analysed the expression of nine structural genes and five regulatory genes in developing embryos of HiII and engineered purple embryo maize. As shown in Figure 1d, among fourteen tested genes, five genes, including structural genes *ZmCHI* and *ZmF3′5′H* and regulatory genes *ZmR1*,* ZmR2* and *ZmVp1*, showed equally high transcript abundance in both HiII maize and transgenic maize (Figure 1d). Four genes (*ZmCHS*,* ZmF3H*,* ZmANS* and *ZmF3′H*) expressed weakly and five genes (*ZmC1*,* ZmIn1*,* ZmDFR*,* ZmBz1* and *ZmBz2*) were not detected in HiII embryos, whereas all the nine genes were actively expressed in purple transgenic embryos (Figure 1d). These results indicate that all the structural genes were intact in the HiII genome, and the difference in transcriptional level of these genes may be responsible for the lack of anthocyanin biosynthesis in HiII embryos. Embryos of transgenic line LX‐4‐2, derived from construct pCL1 that overexpresses only regulatory genes *ZmC1* and *ZmR2*, accumulated as much anthocyanins as LX‐6‐2 and LX‐14‐3, which additionally stacked structural genes *ZmBz1* and *ZmBz2*. The similar expression profiles of endogenous structural genes between LX‐4‐2 and LX‐6‐2 and LX‐14‐3 (Figure 1d) confirmed that the regulatory genes *ZmC1* and *ZmR2* played key roles in rebuilding the anthocyanin biosynthesis pathway in HiII embryos. This conclusion was further confirmed by the purple callus derived from the construct pBD‐C1R2.

**Figure 1:**
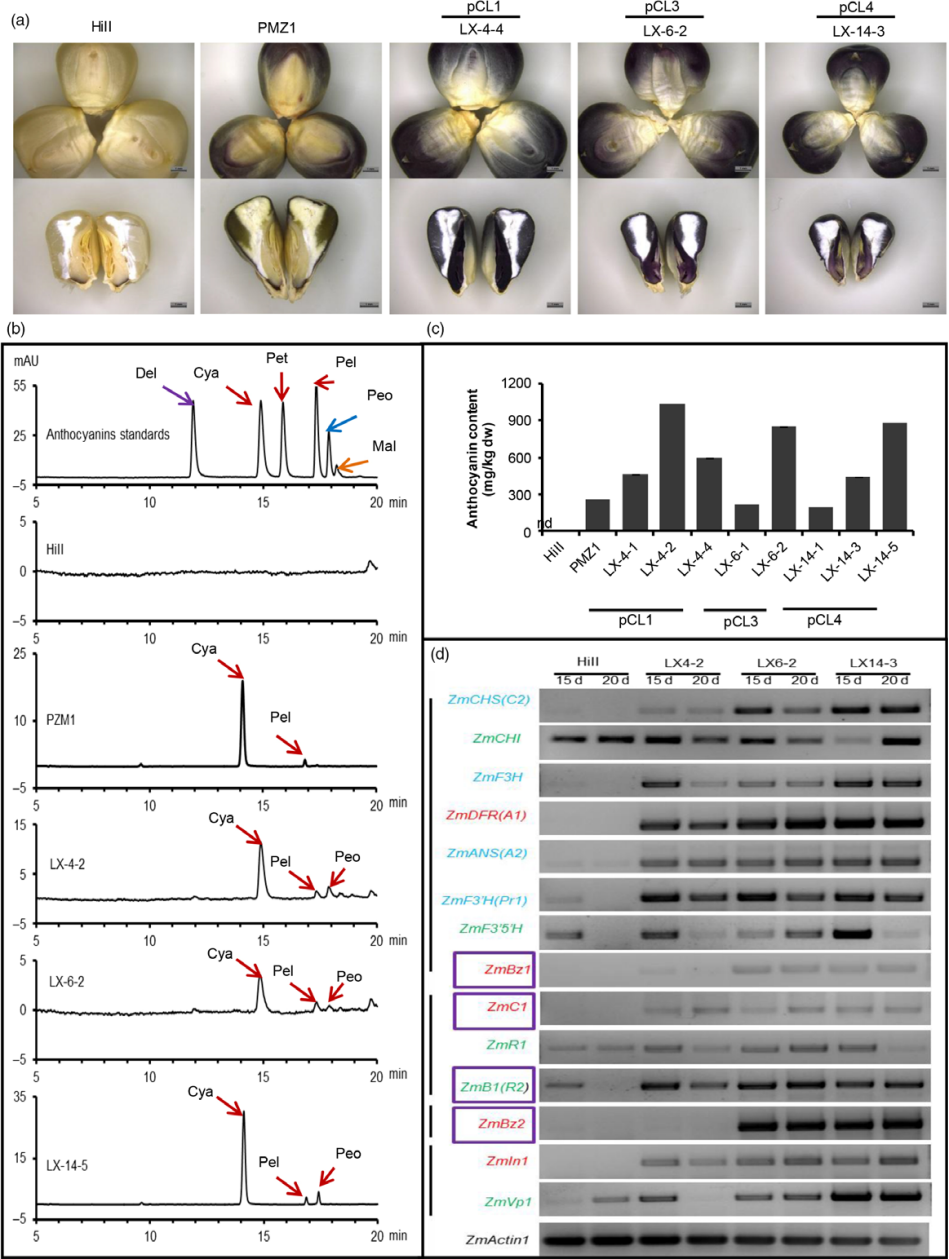
Anthocyanin biosynthesis in the maize embryo and aleurone layer. (a) Kernels from the HiII, the PMZ1 and purple embryo and aleurone layer transgenic maize. (b) HPLC analysis of the major kinds of anthocyanins in kernels. Del: delphinidin; Cya: cyanidin; Pet: petunidin chloride; Pel: pelargonidin; Peo: peonidin; Mal: malvidin. (c) Quantitative analysis of total anthocyanin content in kernels, mg/kg dw, milligrams per kilogram maize kernels dry weight; nd, not detectable. LX‐4‐1/2/4, LX‐6‐1/2 and LX‐14‐1/3/5 are independent events derived from pCL1, pCL3 and pCL4, respectively. Each event has three technical repeats. (d) The expression profiles of anthocyanin biosynthesis‐related genes in HiII and transgenic maize plants. The genes (green) expressed normally, (blue) expressed weakly, (red) not expressed in the developing embryos of HiII, while enhanced or activated in transgenic maize. Purple‐boxed genes are the transgenes; all semiquantitative RT–PCR are 35 cycles, 15d/20d: 15/20 days after pollination.

In this study, we developed a simple and efficient multigene expression system based on an embryo‐specific bidirectional promoter and 2A linker peptides. Eleven genes were successfully introduced into transgenic maize to rebuild the anthocyanin biosynthesis pathway in HiII embryos, resulting in an anthocyanin‐rich purple embryo maize germplasm. This system could be applied in complex metabolic engineering and other genetic improvement and plant biotechnology applications.

## Conflict of interest

The authors declare no conflict of interest.
